# William A. Bronson, M.D., M.D.S.

**Published:** 1890-10

**Authors:** 


### Obituary.

### WILLIAM A. BRONSON, M.D., M.D.S.

Dr. Bronson died at his home, No. 8 East Thirty-fourth Street, New York, on the 20th of August. He was born in Connecticut, June 4, 1817; graduated at Yale in 1840, and commenced the practice of dentistry in 1845.

Dr. Bronson was one of the best esteemed members of his specialty. Honest in every purpose and of unquestioned integrity; faithful in every work; modest and gentle in manner; generous even to a fault; a true friend worthy of the highest confidence, and possessing a most genial and kindly disposition. He was respected and loved by all who knew him, and many will miss his charming companionship and sincerely mourn his departure.

Dr. Bronson was one of the early members of the New York Odontological Society, connecting himself with that body soon after its organization, and taking a most active part in all its proceedings. For two years he held the office of president, and for many years was a member of the Executive Committee. He was one of the founders of the First District Society of New York, and one of the original members of the New York State Dental Society; and in these also he took great interest.

Dr. Bronson was a skilled operator and possessed remarkably good judgment in matters relating to his specialty. He was frequently consulted in cases difficult to diagnosticate or successfully treat, and his professional friends found in him a ready helper and good counsellor. He was a fluent writer. His contributions to the journals and essays read at society meetings were always interesting and instructive. Dr. Bronson was one of nature's noblemen. He was an honor to his profession, and his life was an example well worthy of imitation.

